# Targeting the Heterogeneous Tumour-Associated Macrophages in Hepatocellular Carcinoma

**DOI:** 10.3390/cancers15204977

**Published:** 2023-10-13

**Authors:** Aloña Agirre-Lizaso, Maider Huici-Izagirre, Josu Urretabizkaia-Garmendia, Pedro M. Rodrigues, Jesus M. Banales, Maria J. Perugorria

**Affiliations:** 1 Department of Liver and Gastrointestinal Diseases, Biodonostia Research Institute, Donostia University Hospital, University of the Basque Country (UPV-EHU), 20014 Donostia-San Sebastian, Spain; alona.agirrelizaso@biodonostia.org (A.A.-L.); maider.huiciizagirre@biodonostia.org (M.H.-I.); josu.urretabizkaiagarmendia@biodonostia.org (J.U.-G.); pedromiguel.rodriguesvieira@biodonostia.org (P.M.R.); jesus.banales@biodonostia.org (J.M.B.); 2 Centre for the Study of Liver and Gastrointestinal Diseases (CIBERehd), Instituto de Salud Carlos III (ISCIII), 28029 Madrid, Spain; 3 IKERBASQUE, Basque Foundation for Science, 48009 Bilbao, Spain; 4 Department of Biochemistry and Genetics, School of Sciences, University of Navarra, 31008 Pamplona, Spain; 5 Department of Medicine, Faculty of Medicine and Nursing, University of the Basque Country (UPV/EHU), 20014 Donostia-San Sebastian, Spain

**Keywords:** liver cancer, hepatocellular carcinoma, immunotherapy, tumour microenvironment, tumour-associated macrophages, innate immune system, macrophage reprogramming

## Abstract

**Simple Summary:**

Hepatocellular carcinoma (HCC) is a highly lethal disease with an increasing incidence. Despite the advancements in diagnosis and recent therapeutic options, improving the prognosis of HCC patients remains challenging. One of the reasons of the unsatisfactory outcome of patients with HCC is the complex tumour microenvironment (TME), which is composed of immune and stromal cells, limiting effective treatments. Recent research has highlighted the importance of macrophages in the development and progression of HCC, opening new possibilities for therapy. This review focuses on the heterogeneity of tumour-associated macrophages (TAMs) in HCC, the mechanisms through which HCC tumour cells polarize macrophages, and the therapeutic targets that are currently being tested to explore novel therapies that can improve the prognosis and quality of life of HCC patients.

**Abstract:**

Hepatocellular carcinoma (HCC) is a prevalent and aggressive cancer that comprises a complex tumour microenvironment (TME). Tumour-associated macrophages (TAMs) are one of the most abundant immune cells present in the TME, and play a key role both in the development and in the progression of HCC. Thus, TAM-based immunotherapy has been presented as a promising strategy to complement the currently available therapies for HCC treatment. Among the novel approaches focusing on TAMs, reprogramming their functional state has emerged as a promising option for targeting TAMs as an immunotherapy in combination with the currently available treatment options. Nevertheless, a further understanding of the immunobiology of TAMs is still required. This review synthesizes current insights into the heterogeneous nature of TAMs in HCC and describes the mechanisms behind their pro-tumoural polarization focusing the attention on their interaction with HCC cells. Furthermore, this review underscores the potential involvement of TAMs’ reprogramming in HCC therapy and highlights the urgency of advancing our understanding of these cells within the dynamic landscape of HCC.

## 1. Introduction

Hepatocellular carcinoma (HCC) is the most common form of liver cancer and accounts for ~80% of cases [1]. Currently, HCC is considered a global health concern as its global incidence is increasing, being the third leading cause of cancer death worldwide, with approximately 18% of patients experiencing a relative 5-year survival rate [1,2]. The majority of HCC cases commonly arise in individuals with underlying chronic liver diseases resulting from hepatitis B virus (HBV) or hepatitis C virus (HCV) infection, alcohol abuse and metabolic liver disease, particularly nonalcoholic fatty liver disease (NAFLD) [3]. 

A significant proportion of HCC cases are still diagnosed at advanced stages, compromising the therapeutic options. In this regard, the advancements in the understanding of the molecular biology of the HCC has allowed us to develop molecular targeted agents (MTAs), including immune-checkpoint inhibitors (ICIs) [4,5], tyrosine kinase inhibitors (TKIs) and monoclonal antibodies (mAb) as a useful strategy for the treatment of advanced HCC. Sorafenib was the first treatment approved for HCC [6] and this paved the way for the development of new drugs including multi-targeted TKIs [7,8,9], as well as the vascular endothelial growth factor (VEGF) inhibitor [10]. Importantly, during the last few years, immunotherapy has made considerable progress in HCC treatment and the combination of ICIs and the VEGF inhibitor (atezolizumab plus bevacizumab) is currently the first-line treatment for patients with advanced HCC [11,12]. However, only some patients respond to this treatment and although inflamed and noninflamed HCC tumours and genomic signatures have been described and associated with response to ICIs [13], it is still important to develop a better understanding of the immune landscape of HCC. 

The tumour microenvironment (TME) includes innate and adaptive immune cells, stromal cells, endothelial cells, cancer-associated fibroblasts and the extracellular matrix (ECM). Together, these components create a niche where tumour cells can grow and disseminate [14]. Tumour-associated macrophages (TAMs) are one of the most abundant stromal components in the TME of HCC tumours, and play a key role in promoting tumour progression by enhancing tumour growth, ECM remodeling, angiogenesis and metastasis, as well as in resistance to chemotherapeutic agents and checkpoint blockade immunotherapy [14,15]. Given the multifaceted functions of TAMs in the progression of HCC [15], selectively targeting the immunosuppressive TAMs within the TME holds promise in order to complement, and synergize with, currently available tools for HCC treatment. Novel approaches focusing on TAMs have been used in various strategies including their depletion, inhibition of their recruitment and reprogramming their functional state [16]. In this sense, macrophage reprogramming is devoid of the toxicities that involve strategies such as the ablation of all macrophages [16], and allows us to take advantage of their plasticity to change their phenotype and enhance their anti-tumour capacity. In this review, we discuss the heterogeneity of TAMs in HCC, the mechanisms by which tumour cells reprogram these cells and novel therapeutic options that are currently being tested at the preclinical and clinical levels. 

## 2. Heterogeneity and Plasticity of TAMs in HCC

TAMs are considered a diverse and heterogeneous cell population originating from multiple sources and displaying various phenotypes and functions. Macrophages can be either embryonically seeded in the liver, where they continue to renew themselves, or derived from monocyte precursors that infiltrate tissues and undergo differentiation in response to specific microenvironmental conditions [17]. TAMs are primarily believed to arise from circulating monocytes, which respond to inflammatory signals emitted by tumour cells, leading to their differentiation into TAMs and contributing to tumour progression [18]. However, some studies also suggest that Kupffer cells (KCs) might also account for a small proportion of the total TAM pool of HCC [19,20]. In a study by Sharma et al., three populations of TAMs were identified and named as folate receptor beta (FOLR2) TAM1, osteopontin (SPP1) positive TAM2 and metallothionein 1G (MT1G)-enriched TAM3 [21]. While TAM2 and TAM3 clusters are believed to originate from monocytes, the TAM1 population can be further divided into two clusters. One of these clusters is predicted to be monocyte-derived, while the other cluster shows significant similarity to KCs [21,22]. Moreover, in another study, two macrophage clusters among seven clusters in HBV/HCV-related HCC tumours were considered Kupffer-like cells owing to their high expression of VSIG4, a membrane protein specific to tissue-resident macrophages [20]. 

Traditionally, macrophages have been categorized into two different activation states based on the expression of cell surface polarization markers as classically activated (M1) and alternatively activated (M2). TAMs are generally classified as pro-tumourigenic M2-like macrophages and their presence has been associated with a worse clinical outcome [20]. In this regard, a M2-like TAM-related signature associated with a poor prognosis in HCC patients has been described including prognosis-related genes such as PDLIM3, PAM, PDLIM7, FSCN1, DPYSL2, ARID5B, LGALS3, and KLF2 [23]. However, recent studies employing single-cell RNA-sequencing (sc-RNA-seq) technology have revealed the heterogeneity of TAMs in HCC. These studies have shown that certain TAM populations express both M1 and M2 markers, indicating a spectrum of intermediary phenotypes and highlighting their pleiotropic functions [20,24,25]. Thus, TAMs represent a highly heterogeneous cell population that can exert either pro-tumour or sometimes anti-tumour activities. Indeed, Song et al. identified a IL1B^+^ macrophage cluster in patients with HBV/HCV-related HCC that might be involved in anti-tumour responses, although further experiments need to be conducted to confirm this statement [20]. 

Multiple single-cell atlases of the HCC TME have been conducted using sc-RNA-seq technology and revealed the distinct transcriptional landscapes of each subpopulation within the TME [20,21,24,25,26,27,28,29,30]. These studies have also highlighted a significant level of heterogeneity among macrophages across different tumours, as certain populations were found to be specifically associated with individual patients, indicating inter-tumoural heterogeneity [27]. Importantly, it should be noted that each study has employed its own unique nomenclature for assigning names to the various subpopulations based on genes that were highly expressed in each specific subpopulation. However, it is important to note that there is considerable overlap among the different studies conducted, as shown in Table 1. This indicates that despite the unique nomenclature applied by each study, there are shared characteristics and subpopulations across multiple single-cell atlases of the HCC TME. 

In a study by Lu et al. [27], five macrophage clusters enriched in tumour tissues were identified, which were consistent across patients. These clusters included anti-inflammatory TREM2^+^ macrophages newly recruited into tumours, which shared similarities with the FOLR2^+^ TAM1 cluster from Sharma’s HCC dataset [21] and Liu et al.’s FOLR2^+^ cluster [26]. Additionally, Lu et al. also described monocyte-derived FCN1^+^ LYZ^+^ VCAN^+^ macrophages, VEGFA^+^ macrophages associated with oxidative stress, and MMP9^+^ SSP1^+^ macrophages, which were considered terminally differentiated TAMs. The latter TAMs were believed to originate from monocyte-derived FCN1^+^ LYZ^+^ VCAN^+^ or TREM2^+^ macrophages and promote HCC cell migration, invasion, and tumour angiogenesis. These MMP9^+^ SSP1^+^ macrophages shared some similarities with SPP1^+^ TAM2s expressing TREM2 in Sharma’s HCC dataset [21,27]. Furthermore, Gao et al. [28] observed that compared to adjacent non-tumoural liver tissue, the APOC1^+^ SSP1^+^ TAM and HSPA1B^+^ TAM subpopulations were increased in tumour tissue. However, it remains uncertain and requires further confirmation whether these APOC1^+^ SPP1^+^ TAMs identified by Gao et al. represent the same population previously defined by Sharma et al. and Lu et al. [21,27]. Interestingly, recently, Liu et al. combined spatial transcriptomics with sc-RNA-seq and found that SPP1^+^ macrophages interact with cancer-associated fibroblasts (CAFs) to form a spatial structure named the tumour immune barrier (TIB) that limits the infiltration of immune cells into the tumour core [26]. 

**Table 1:** Marker genes for macrophage clusters from single-cell atlases of the HCC TME.

Zhang et al. [25]									
Mφ-THBS1^+^	Mφ-C1QA^+^		Mφ-APOE^+^		Mφ-GPX3^+^		Mφ-VCAN^+^		Mφ-MARCO^+^
CST3, LYZ, CD68, THBS1, S100A8, S100A9	CST3, LYZ, CD68, CD163, CD169, C1QA, SLC40A1, GPNMB		CST3, LYZ, CD68, CD163, CD169, APOE		CST3, LYZ, CD68, FCGR3A, SAT1, LST1, HLA-DQB1		CST3, LYZ, CD68, S100A4, HLA-DRA, HLA-DQA1		CST3, LYZ, CD68, MARCO, S100A9, CD163
Sharma et al. [21]									
TAM 1			TAM 2				TAM 3		
C1QC, C1QA, SEPP1, LGMN, APOC1, CTSD, CD68, MS4A4A, PLD3, FOLR2, GPNMB, CD63, CTSB, FCGR3A, PSAP, FTL, CD14, LIPA, TYROBP, NPC2, DAB2, FCGRT, RNASE1, CTSC, FCER1G, C1QB, SLC40A1, APOE, CCL3, CCL3L3, MS4A7, CCL4L2, CD5L, VCAM1, CST3, SDC3, ITM2B, CCL4, CXCL2, IGF1, CD163, HES1, FOS, IER3			RNASE1, CTSD, CSTB, NUPR1, FTL, APOC1, GPNMB, CTSB, APOE, CTSZ, LGALS1, PLTP, FABP5, TREM2, CXCL3, COLEC12, HSPA1A, PLIN2, SCD, CTSL, HSPA1B, MMP19, PLD3, ABL2, CEBPB				MT1H, MT1X, MT1M, MT1E, MT1F, APOC3, ORM1, FTL, APOA2, APOC1, MT1A, ALB, ORM2, MT2A, AMBP, APOA1, AHSG, SAA1, RBP4, SIRPG, KNG1, LINC00520, OTOA, UCHL1, APOB		
Song et al. [20]									
Mφ-IL1B^+^		Mφ-VSIG4^+^		Mφ-FABP5^+^		Mφ-S100A6^+^		Mφ-S100A12^+^	
TNFAIP3, IL1B, HSPA1B, AREG, CXCL10, WARS, CCL3L1, DNAJB1, GBP1, APOB, C3A, EREG, GBP5, G0S2, CXCL9		GLDN, VSIG4, ADAMTS2, RPS26, TREM2, ALB, APOC3, RNASE1, SAA1, VWF, APOA1, GPR34, MS4A4A, STAB1, MSR1, SLCO2B1, A2M, HP, SELENOP, ABCC5, FOLR3, AL355075.4, FCER2, CLEC5A, OLR1, ALOX5AP, FN1, MT1G		FABP4, PDK4, DHRS9, CXCL12, LIPA, ATP1B1, CLEC10A, MGLL, NRP1, ITGB5, MMP9, IGF1, TIMP3, SCN1B, HRH1, WWP1, FABP5, LPL, VCAM1, LILRB5, MATK, EGFL7, PHLDA1, CCL2, TM4SF19		AL391807.1, S100A8, S100A9, VCAN, SULT1A1, RETN, CCR2, CDA, NRG1, CYP1B1, SELL, IL17RA, S1PR3, POU2F2, RFLNB, IGHA1, F5, AC007952.4, MGST1, CX3CR1		MNDA, S100A8, S100A12, NFE2, CDA, CRISPLD2, TMEM154, SHROOM1, S1PR3, PRAM1, SLC46A2, NLRP12, LFNG, CX3CR1, NHSL2, S100Z	
Mφ-CCL18^+^					Mφ-AQP9^+^				
CCL18, NUPR1, SCD, GATM, PLTP, GPNMB, APOC1, LGMN, RGS1, ARL4C, ATP6V0D2, APOE, SELENOP, A2M, PMP22, CTSB, CREM, SLC40A1, IGF1, CTSL, PLXDC1, ABCA1, DAB2, SPP1, C2, SLCO2B1, MSR1, CD163L1, SDC3, ABCG1, SLC2A1, CD59, FOLR2, RARRES1, AVPI1, RNASE1, FABP3, SLC7A8, ELL2, TNFRSF21, PLD3, HLA-DQA1, FUCA1, ACP5, CTSD, CD28, SMPDL3A, ENPP2, NRP1, IL2RA, ANKRD37, TSC22D1, OTOA, NPL, KCNMA1, SLC2A5, FABP5, ZNF331, AKR1B1, PLA2G7, ME1, VAT1, CSTB, SDS, SLC19A2, COLEC12, GAL3ST4, TFRC, ADM, ANKH, NRP2, FRMD4A, GPR137B, SGPL1, PKD2L1, HS3ST2, TCEAL9, MMP19, SDC2, BEX3, HSD17B14, CD209, ARRDC3, IL18BP, FARP1, EPHX1, VCAM1, SLC16A10, BNIP3, HLA-DRB5, FAM213A, GPX3, MAFF, CXCL3, RAB42, TMIGD3, CHCHD6, IGSF21, MMP14, CXCL2, INSIG1, EGLN3, ZNF395, ADAMDEC1, ADAM8, CHMP1B, CD5L, PLAU, HILPDA, HK2, GADD45A, SERPINF1, CXCL8, IL4I1, CCL4L2					AQP9, VCAN, SLC11A1, IFI44L, S100A12, NAMPT, FPR1, S100A8, CD55, IFI6, LILRA5, S100A9, IFITM2, VNN2, AC245128.3, SOCS3, CD300E, VSTM1, SLC25A37, MX2, ACSL1, IFITM3, LY6E, HMGB2, PLSCR1, PHC2, NLRP3, CLEC4E, ISG15, SLC2A3, SERPINB1, MX1, SELL, IFI44, ICAM3, FPR2, TREM1, THBS1, NFIL3, CLU, TIMP1, EREG, IRF7, UPP1, RIPOR2, GCA, RETN, MEGF9, CLEC4D, XAF1, MCTP2, OSCAR, LRRK2, MXD1, NFE2, RNASE2, CDA, AGFG1, OAS2, LINC02207, PGD, CRISPLD2, IL1R2, FES, CDKN2D, AL034397.3, CPD, AREG, GK, PROK2, FYN, LINC00937, MPHOSPH6, ECE1, CCDC69, RSAD2, RAB27A, CKAP4, OASL, MBOAT7, TMEM71, CR1, PADI4, MCEMP1, F5, CRIP1, CREB5, IL1RAP, MFGE8, GK5, CYP1B1, HBEGF, THBD, LTB4R, HERC5, ANXA6, FCAR, ADM, PLBD1, PDE4D, SPATA13, CEACAM4, MAN2A2, OAS3, VNN1, BCL3, TSPAN2, PTGER2, OSM, SPRY1, TOR1B, TOB1, TRMT6				
Sun et al. [24]									
Mφ-c1		Mφ-c2		Mφ-c3		Mφ-c4		Mφ-c5	
CD163, CD68, SLC40A1, SELENOP, FOLR2, APOA2, IGLL5		SLC16A10, CTNNB1, WTAP, SERINC5, PHACTR1		SPP1, CSTB, RNASE1, MMP12, HK2		BAG3, HSPB1, ZFAND2A, HSPH1, HSPA6, DNAJA4		APOA2, BAG3, HSPB1, ZFAND2A, HSPH1, HSPA6, DNAJA4	
Lu et al. [27]									
Mφ-MARCO^+^	Mφ-TREM2^+^		MoMFs-c1		MoMFs-c2		Mφ-VEGFA^+^		Mφ-MMP9^+^
MARCO, CD5L, C1QB, C1QA, SLC40A1, LIPA, MS4A7, MS4A6A, CFD, CD163	C1QC, C1QB, C1QA, RNASE1, LGMN, HLA-DRA, MS4A4A, HLA-DPA1, SLC40A1, CD74		S100A8, S100A9, IL1B, CXCL8, CXCL3, G0S2, EREG, PLAUR, CCL20, CXCL2		FCN1, S100A8, S100A9, G0S2, LST1, BCL2A1, AIF1, LYZ, BAG3, C15orf48		RNASE1, CCL3L3, CCL4L2, C1QA, LGMN, CCL3L3, CEBPB, CD83, BAG3, CCL18		SSP1, MMP12, MMP9, FABP5, CSTB, GPNMB, LGALS1, RNASE1, C15orf48, CXCL8
Liu et al. [26]									
Mφ-SPP1^+^									
CSTB, SPP1, FTL, FABP5, CTSD, RNASE1, GPNMB, LGALS1, TM4SF19, NUPR1, CTSL, LGALS3, CCL7, VIM, SLAMF9, FABP4, BNIP3, MIF, ATP6V1F, CD68									

In addition, Zhang et al. found two distinct macrophage clusters enriched in tumour tissues. These clusters were THBS1^+^ macrophages, which exhibited a signature similar to myeloid-derived suppressor cells (MDSCs) and were thus referred to as MDSC-like macrophages, and C1QA^+^ macrophages, which simultaneously presented signatures for TAMs, M1, and M2 macrophages [25]. Interestingly, MDSC-like macrophages highly expressed the S100A family genes FCN1 and VCAN, suggesting these could be the monocyte-derived macrophages also described by Lu et al. and Song et al. [20,27], and the C1QA^+^ macrophages highly expressed other genes such as APOE and TREM2. Additionally, Song et al. found a macrophage population that was absent in previous sc-RNA-seq studies and was mostly infiltrated in advanced HCC patients, and characterized by a higher expression of CCL18 [20]. These macrophages showed strong activity in lipid transport and metabolism, and immunosuppressive-related pathways. 

Besides the heterogeneity observed at the individual cell level, it has been demonstrated that immune cell infiltrates exhibit notable differences between intrahepatic metastatic lesions in multifocal HCC and those in cases of multicentric occurrence. Indeed, in metastases, a higher presence of M2 macrophages and a lower abundance of T cells have been observed [31]. However, further studies need to be performed to characterize the distinct transcriptional landscapes. 

## 3. The Dynamic Crosstalk between HCC Tumour Cells and TAMs

The TME, and especially TAMs, orchestrate complex and dynamic interactions with HCC tumour cells by cell-to-cell contact and/or soluble messengers [32]. In this regard, HCC tumour cells recruit monocytes to the HCC TME by secreting various factors and promote the transition into TAMs with a predominant M2-like phenotype [33]. Likewise, TAMs interact with the neighbouring cells in order to maintain an immunosuppressive microenvironment that ultimately results in tumour development and progression [34]. 

### 3.1. The Effect of HCC Tumour Cells Inducing M2 Polarization of TAMs in the TME

HCC tumour cells interact with TAMs through the secretion of diverse signalling molecules and exosomes that contain proteins, metabolites and nucleic acids (Table 2). 

#### 3.1.1. Signalling-Molecule Mediated Crosstalk

Cytokines represent a group of signalling proteins that play key roles in the immunomodulation of cancer [35]. In HCC, the interleukin-8 (IL-8) secreted by tumour cells induces the M2 polarization of TAMs, which further contributes to the epithelial to mesenchymal transition (EMT) of HCC cells [36]. On the other hand, the secretion of the pleiotropic cytokine IL-6 by HCC cells promotes the recruitment of TAMs to the TME, with the expression levels of this cytokine being associated with a poor prognosis of the patients [37]. Moreover, IL-6 secreted by HCC cells was shown to induce the expression of the immune checkpoint molecules such as programmed cell death ligand 1 (PD-L1) in TAMs and thus modulate immunosuppression [38]. Importantly, understanding the mechanisms involved in the regulation of immunosuppressive molecules could help to improve the efficacy of ICIs. Other pro-inflamatory cytokines such as IL-1β were shown to upregulate PD-L1 and colony-stimulating factor 1 (CSF1) expression in HCC cells [39]. HCC-derived CSF1 shifts macrophage polarization toward an M2 phenotype [40]. Indeed, it has been shown that inhibiting CSF1 receptor halts HCC progression in mouse models by inducing TAM polarization towards an M1-like phenotype [41]. Other cytokines such as osteopontin (SPP1) have also been associated with an immunosuppressive phenotype of macrophages in HCC. SPP1 facilitates M2-like polarization of macrophages, and promotes the expression of PD-L1 in HCC cells through the activation of the CSF1/CSF1R pathway in macrophages [42]. Moreover, multiomics analysis demonstrated that the oncogene SPP1 was associated with a poor prognosis in HCC. In vitro assays further demonstrated that SPP1 mediates interactions between HCC cells and TAMs by acting as a ligand, promoting M2-like polarization [43]. In addition, the chemokine CCL5 secreted by HCC tumour cells enhances the M2/M1 ratio of macrophages, boosting HCC progression [44]. Drp1-mediated mitochondrial fission has been reported to induce the cytosolic mitochondrial DNA (mtDNA) stress in HCC cells, which enhances CCL2 secretion by the TLR9-mediated NF-kB signalling pathway and promotes TAM recruitment and polarization [45]. Apart from CCL2, other HCC-derived cytokines and growth factors such as the macrophage inhibitory factor (MIF) and hepatocyte growth factor (HGF) have been reported to promote macrophage recruitment and M2-like stimulation [46,47]. In line with this, miR-144/451a cluster silencing by epigenetic mechanisms was shown to contribute to HCC progression targeting MIF and HGF [48].

Regarding specific signalling pathways, it has been demonstrated that HCC tumour cell-derived WNT ligands interact with macrophages, inducing polarization into an immunosuppressive M2-like phenotype, which in turn leads to tumour growth, migration and metastasis [49]. Likewise, several studies have emphasized the relevance of WNT and other major developmental signalling pathways (e.g., NOTCH) on TAM differentiation and consequent HCC progression, opening the door for new treatments targeting the WNT pathway [19,50,51]. 

Other signalling molecules that are secreted by HCC tumour cells include the protein high mobility group box 1 (HMGB1), which promotes autophagy-regulated M2 polarization of TAMs via the production of ROS in these cells through the TLR2/NOX2 axis [52]. In vivo assays with HCC-bearing mice showed that by inhibiting HMGB1 and ROS, M2-like TAM accumulation and nodule formation decreased [52]. 

On the other hand, the secretion of reactive nitrogen species (RNS) such as nitric oxide (NO), which were shown to be regulated by the transcription factor forkhead box O-1 (FOXO1) in HCC cells, modulates TAM infiltration and polarization towards an antitumour phenotype by reducing IL-6 and CD206 expression in these macrophages [53]. 

#### 3.1.2. Exosome-Mediated Crosstalk

In the context of HCC, tumour cell-derived exosomes are known to modulate macrophage polarization and promote immune escape and tumour progression [54,55]. Among the transported molecules, noncoding RNA molecules (e.g., microRNAs, circular RNAs and long noncoding RNAs) constitute a major group and act as oncogenes or tumour suppressors [56]. In line with this, HCC tumour cell-derived exosomal miR-21-5p promotes TAM differentiation into an M2 phenotype by directly targeting Ras homolog family member B (RhoB) [57]. Likewise, exosomal miR-23a-3p released from endoplasmatic reticulum-stressed HCC cells has been reported to induce PD-L1 expression in TAMs and consequently inhibit T cell function [58]. Regarding the abovementioned relevance of PD-L1/PD-1 in maintaining an immunosuppressive TME, another study described how GOLM1-mediated exosomal PD-L1 transport into TAMs aggravated CD8^+^ T cell suppression in HCC [59]. Additionally, exosomal miR-146a-5p has also demonstrated to drive the M2 polarization of TAMs and disrupt T cell functions [60]. Moreover, a study by Zongqiang et al. described that HCC cell-derived exosomal miR452-5p targets TIMP3 in TAMs, provoking M2 polarization and fostering HCC progression [61]. Besides miRNAs, circular RNAs play crucial roles in tumour immunity by acting as miRNA sponges, among others [62]. An example of that could be circTMEM181, which was found to be upregulated in anti-PD1 therapy-resistant HCC patients [63]. Mechanistically, exosomal circTMEM181 sponges miR-488-3p and enhances CD39 expression in macrophages, leading to CD8^+^ T cell dysfunction and anti-PD-1 resistance. Furthermore, a recent study shows that tumour-derived exosomal hsa_circ_0074854 participates in the differentiation of M2-like TAMs and its suppression inhibits HCC tumour growth [64]. 

**Table 2:** HCC tumour cell-derived molecules that participate in the crosstalk with TAMs.

Molecule	Type of Crosstalk	Mechanism of Action and Effect on TAMs	References
IL-8	Signalling molecule-mediated	- Induces polarization of TAMs into a M2 phenotype, further contributing to the EMT of HCC cells.	[36]
IL-6	Signalling molecule-mediated	- Promotes the recruitment of TAMs to the TME. - Induces expression of PD-L1 in HCC TAMs.	[37,38]
CSF1	Signalling molecule-mediated	- Induces M2-like polarization of macrophages and anti-PD-1 resistance.	[40,41]
SPP1	Signalling molecule-mediated	- Promotes M2-like polarization of macrophages.	[43]
CCL5	Signalling molecule-mediated	- Enhances the M2/M1 ratio of macrophages, boosting HCC progression.	[44]
CCL2	Signalling molecule-mediated	- Promotes TAM recruitment and polarization.	[45]
Acetoacetate	Signalling molecule-mediated	- Induces TAM recruitment and enhances M2 polarization of macrophages by regulating MIF activity.	[46,48]
HGF	Signalling molecule-mediated	- Induces migration and regulates the distribution of M2 macrophages in the tumour tissue.	[47,48]
WNT ligands	Signalling molecule-mediated	- Induce polarization of macrophages into an immunosuppressive M2-like phenotype, which in turn leads to tumour growth, migration and metastasis.	[49]
HMGB1	Signalling molecule-mediated	- Promotes M2 polarization of TAMs via the HMGB1/TLR2/NOX2/autophagy axis.	[52]
NO	Signalling molecule-mediated	- Enhances TAM infiltration and polarization towards an anti-tumour phenotype. - Reduces IL-6 and CD206 expression in macrophages.	[53]
miR-21-5p	Exosome-mediated	- Promotes TAM differentiation into a M2 phenotype via RhoB UTR targeting.	[57]
miR-23a-3p	Exosome-mediated	- Induces PD-L1 expression in TAMs and consequently inhibits T cell function.	[58]
PD-L1	Exosome-mediated	- Aggravates CD8+ T cell suppression in HCC.	[59]
miR-146a-5p	Exosome-mediated	- Drives M2 polarization of TAMs and disrupts T cell functions.	[60]
miR452-5p	Exosome-mediated	- Targets TIMP3 in TAMs, provoking a M2 polarization and fostering HCC progression.	[61]
circTMEM181	Exosome-mediated	- Sponges miR-488-3p and enhances CD39 expression in macrophages, leading to CD8+ T cell dysfunction and anti-PD-1 resistance.	[63]
hsa_circ_0074854	Exosome-mediated	- Participates in the differentiation of M2-like TAMs and its suppression inhibits HCC tumour growth.	[64]

Abbreviations: CCL, C-C motif chemokine; CD, cluster of differentiation; CSF1, colony-stimulating factor 1; EMT, epithelial to mesenchymal transition; HCC, hepatocellular carcinoma; HGF, hepatocyte growth factor; HMGB1, high mobility group box 1; IL, interleukin; MIF, macrophage inhibitory factor; NO, nitric oxide; NOX2, NADPH oxidase 2; PD-1, programmed cell death protein 1; PD-L1, programmed cell death ligand 1; RhoB, Ras homolog family member B; SPP1, osteopontin; TAMs, tumour-associated macrophages; TIMP3, metalloproteinase inhibitor 3; TLR2, toll-like receptor 2; TME, tumour microenvironment; UTR, untranslated region; WNT, wingless-related integration site.

### 3.2. The Effect of M2-Like TAMs Promoting the Progression, Growth and Invasiveness of HCC

In recent years, many studies have aimed to elucidate the molecular mechanisms that hide behind the complex interactions of M2-like TAMs with tumour cells favouring HCC progression (Table 3). 

#### 3.2.1. Signalling Molecule-Mediated Crosstalk

In respect of signalling molecule-mediated crosstalk, it has been shown that in hypoxic and inflammatory conditions, M2-like TAMs secrete high amounts of IL-1β, inducing an overexpression of hypoxia inducible factor (HIF)-1α in HCC cells, the latter being responsible of an increased EMT of HCC cells and HCC metastasis in mouse models [65]. Moreover, numerous works have underlined the importance of TAMs-derived IL-6 in promoting HCC progression and metastasis [66,67,68].

Similarly, IL-8 has been shown to promote EMT of HCC tumour cells via the JAK2/STAT3/Snail signalling pathway, and hence induce their migration and invasion [69]. In addition, immunosuppressive TAMs have shown to promote EMT on HCC cells via WNT/β catenin pathway by releasing tumour necrosis factor α (TNF-α) [70]. M2 macrophage-derived chemokine ligand 17 (CCL17) is also known to promote EMT in HCC cells via the WNT/β catenin pathway, contributing to tumourigenesis [71]. 

Besides cytokines, the secretion of TGF-β by TAMs promotes EMT and confers stem-like properties to HCC tumour cells [72]. Likewise, S100 calcium-binding protein A9 (S100A9) secreted by TAMs boosts HCC progression by inducing stemness of tumour cells [73]. In addition, the pro-tumour lectin galectin-1 (Gal-1), when secreted by TAMs via TLR2-mediated secretory autophagy, has been shown to induce HCC progression [74]. Computational analyses of public datasets and in vitro and in vivo experiments have identified the oncoprotein-induced transcript 3 (OIT3) as a novel M2 marker. The overexpression of OIT3 in M2 macrophages was found to be associated with a higher expression of molecules that induce HCC progression, metastasis and immunosuppression such as matrix metallopeptidase 2 (MMP2), VEGFA and PD-L1 in TAMs [75,76]. 

#### 3.2.2. Exosome-Mediated Crosstalk

Exosome-mediated crosstalk is also involved in the interplay between M2 TAMs and HCC tumour cells. Exosomal miR-17-92 clusters from M2-like TAMs were shown to induce a mismatch in the TGF-β1/BMP-7 pathway of HCC tumour cells by modulating post-transcriptional and post-translational modifications of various proteins. As a consequence, miR-17-92 increased HCC tumour cells’ stem-like properties and their invasive capacity [77]. Another M2 macrophage-derived exosomal miRNA with the capacity of stimulating the stemness of HCC cells is miR-27a-3p, which targets thioredoxin-interacting protein (TXNIP) [78]. Likewise, exosomal miR-92a-2-5p secreted by macrophages boosts the invasiveness of tumour cells by modulating the AR/PHLPP/p-AKT/β-catenin signalling pathway [79]. Moreover, miR-660-5p secreted from M2 macrophages modulates Kruppel-like factor 3 (KLF3) in HCC tumour cells, therefore inducing HCC progression [80]. TAMs can also secrete exosomes containing M2 macrophage polarization-associated lncRNAs, such as lncMMPA, which induces the proliferation of HCC tumour cells by regulating their metabolism [81]. On the other hand, exosome-mediated crosstalk also happens between M1 macrophages and tumour cells. For instance, exosomal miR-628-5p secreted by M1 macrophages inhibits HCC progression by targeting the circFUT8/miR-552-3p/CHMP4B pathway [82]. 

**Table 3:** TAM-derived molecules that participate in the crosstalk with HCC tumour cells.

Molecule	Type of Crosstalk	Mechanism of Action and Effect on HCC Tumour Cells	References
IL-1β	Signalling molecule-mediated	- Stimulates an increase in EMT of HCC cells and metastasis via HIF-1α/IL-1β signalling pathway.	[65]
IL-6	Signalling molecule-mediated	- Induces HCC carcinogenesis via STAT3 signalling pathway. - Enhances inflammation response inducing HCC progression. - Stimulates HCC metastasis under hypoxic conditions.	[66,67,68]
IL-8	Signalling molecule-mediated	- Promotes EMT of HCC tumour cells via JAK2/STAT3/Snail signalling pathway and hence induces their migration and invasion.	[69]
TNF-α	Signalling molecule-mediated	- Promotes EMT on HCC cells via WNT/β catenin pathway.	[70]
CCL17	Signalling molecule-mediated	- Promotes EMT on HCC cells via WNT/β catenin pathway, contributing to tumourigenesis.	[71]
TGF-β	Signalling molecule-mediated	- Promotes EMT and confers stem-like properties to HCC tumour cells.	[72]
S100A9	Signalling molecule-mediated	- Boosts HCC progression by inducing stemness of tumour cells.	[73]
Gal-1	Signalling molecule-mediated	- It is secreted by TAMs via TLR2-mediated secretory autophagy and induces HCC progression.	[74]
miR-17-92 cluster	Exosome-mediated	- Induces a mismatch in the TGF-β1/BMP-7 pathway of HCC tumour cells, leading to an increase in HCC tumour cells’ stem-like properties and invasive capacity.	[77]
miR-27a-3p	Exosome-mediated	- Stimulates stemness of tumour cells by targeting TXNIP.	[78]
miR-92a-2-5p	Exosome-mediated	- Boosts invasiveness of tumour cells by modulating AR/PHLPP/p-AKT/β-catenin signalling pathway.	[79]
miR-660-5p	Exosome-mediated	- Modulates KLF3 in HCC tumour cells, therefore inducing HCC progression.	[80]
lncMMPA	Exosome-mediated	- Induces proliferation of HCC tumour cells by regulating their metabolism.	[81]
miR-628-5p	Exosome-mediated	- Inhibits HCC progression by targeting the circFUT8/miR-552-3p/CHMP4B pathway.	[82]

Abbreviations: AR, androgen receptor; BMP-7, bone morphogenetic protein 7; CCL17, C-C motif chemokine 17; CHMP4B, charged multivesicular body protein 4B; EMT, epithelial to mesenchymal transition; Gal-1, galectin-1; HCC, hepatocellular carcinoma; HIF-1α, hypoxia inducible factor 1α; IL, interleukin; JAK2, Janus kinase 2; KLF3, Kruppel-like factor3; PHLPP, PH domain leucine-rich repeat protein phosphatase; S100A9, S100 calcium-binding protein A9; STAT3, signal transducer and activator of transcription 3; TAMs, tumour-associated macrophages; TGF-β, transforming growth factor β; TLR2, toll-like receptor 2; TNF-α, tumour necrosis factor α; TXNIP, thioredoxin-interacting protein; WNT, wingless-related integration site.

## 4. Current Strategies Targeting TAMs in HCC

During the last few years, several preclinical and clinical studies have started to evaluate TAM-centered therapeutic strategies in HCC. In this regard, several approaches for targeting TAMs have been proposed for limiting liver cancer progression, including targeting TAM recruitment and accumulation in the tumour and the functional reprogramming of TAMs (Table 4). 

Although the inhibition of monocyte/macrophage recruitment by chemokine targeting approaches such as blocking the CCL2/CCR2 axis with a CCR2 antagonist suppresses tumour growth [83] and potentiates the therapeutic effects of sorafenib in mouse models of HCC [84], using CCR2 antagonists as a monotherapy might not be as effective as initially thought, as compensatory TAM subsets such as KC-like TAMs can emerge [19]. These results highlight that combination therapies targeting different cell types or the reprogramming of TAMs might be a more viable therapeutic strategy for patients with HCC. Indeed, a clinical trial (NCT04123379) to assess the clinical efficacy of BMS-813160, a CCR2/CCR5 inhibitor, in combination with nivolumab (anti-PD1 mAb) is currently ongoing. In this phase IIa trial designed to assess the clinical efficacy of BMS-813160, patients will receive nivolumab monotherapy or neoadjuvant nivolumab in combination with BMS-813160 prior to surgical resection, with the primary endpoint being significant tumour necrosis (>70% tumour necrosis at time of surgery) and the secondary endpoints being the time to surgery, safety and tolerability, radiographic response, progression-free survival, and overall survival. Another well-stablished method of reducing TAM recruitment and their polarization includes blocking the CSF1/CSFR1 axis, which is a well-known monocyte/macrophage differentiation and survival factor [85,86]. HCC-derived CSF1, which is transcriptionally regulated by ZFP64, induces M2-like polarization of macrophages and anti-PD1 resistance [40]. Mechanistically, blocking the PKCα/ZFP64/CSF1 axis with multi-kinase inhibitors such as Lenvatinib, which is able to decrease PKC expression levels [87], improves anti-PD1 efficacy [40,88]. Other strategies targeting the CSF1/CSFR1 axis like CSF1R blockade by PLX3397, an inhibitor for CSF1R tyrosine kinase, have shown to inhibit tumour progression in mouse models of HCC by shifting the polarization of TAMs [41]. In line with these results, inhibition of the CSF1/CSFR1 pathway reduces macrophage recruitment and M2 phenotype polarization and sensitizes HCC tumours to anti-PD-L1 blockade [42]. These preclinical studies suggest that therapeutic strategies blocking CSF1 signalling via CSFR1 inhibition on TAMs might increase the efficacy of ICIs in patients with HCC. In fact, a phase 2 clinical trial (NCT04050462) with the anti-CSFR1 antibody cabiralizumab as an adjuvant therapy for immune checkpoint-based treatment with nivolumab is currently ongoing in patients with HCC, which aims to measure the objective response rate (ORR) of cabiralizumab in combination with nivolumab in comparison to nivolumab monotherapy.

Although strategies directed to target TAMs have initially been focused on macrophage depletion, there is growing evidence that reprogramming of TAMs to overcome an immunosuppressive environment may constitute a more effective therapeutic strategy in cancer [89]. One of the hotspots in reversing the TAM phenotype in cancer has been inhibiting the phosphoinositide 3-kinase gamma (PI3Kγ), which was identified as a potent regulator of macrophage polarization, stimulating C/EBPβ activation with the consequent activation of an immunosuppressive transcriptional program in various solid tumours [90]. Moreover, it was shown that PI3Kγ inhibition can synergize with T cell-targeted therapy (anti-PD-1) in mouse tumour models [90]. As some of the mechanisms of resistance to sorafenib, which has been the first-line treatment for HCC for many years, have been linked to the presence of M2-type macrophages in HCC [47,91,92], combination strategies such as sorafenib in combination with the PI3Kγ inhibitor (TG100-115) have been tested in the preclinical setting [93]. In line with this, a clinical trial for HCC patients with the combination of a Pan-PI3K inhibitor (SF1126) with the anti-PD1 mAb nivolumab was also designed (NCT03059147). Directly targeting transcription factors such as C/EBPα that regulate the function of myeloid cells and that are deregulated in solid tumours such as HCC [94] can represent another therapeutic option [95]. In this regard, therapeutic upregulation of C/EBPα with small activating RNA (saRNA; MTL-CEBPA) has been tested in the preclinical and clinical setting in combination with sorafenib (NCT02716012), resulting in an overall reduction in pro-tumour M2 TAMs and showing anti-tumour responses in patients with advanced HCC. The anti-tumour effects of MTL-CEBPA in different mouse cancer models were accentuated when this treatment was combined with ICIs [95], with this being the rationale behind designing a new clinical trial that investigates the potential benefit of MTL-CEBPA and pembrolizumab (anti-PD1) combination in patients with liver cancer (NCT04105335). Other protein kinases that are enriched in TAMs of HCC tumours based on single-cell RNA sequencing data include glycogen synthase kinase 3β (GSK3β) [96]. Of note, macrophage GSK3β deficiency halts the progression of HCC by inhibiting the M2 phenotype of TAMs and enhances the sensitivity of anti-PD1 immunotherapy. Inhibiting GSK3β with a GSK3β inhibitor in TAMs reduces the proliferation, migration and invasion of HCC cells in vitro and inhibits tumour growth in vivo improving the sensitivity of the anti-PD1 therapy.

Metabolic reprogramming of TAMs is also linked to the plasticity of these cells. In this respect, it has been shown that receptor-interacting protein kinase 3 (RIPK3) is downregulated in HCC-associated TAMs, its deficiency being associated with a reduction in reactive oxygen species (ROS) production and fatty acid oxidation (FAO) via PPAR pathway activation and an induction of M2 polarization of TAMs [97]. In line with this, FAO blockade in TAMs reverses their immunosuppressive phenotype in mouse HCC tissues and supresses HCC tumourigenesis.

Other strategies of promoting reprogramming of TAMs towards an M1 phenotype includes the use of TLR agonists, such as resiquimod (R848), a potent agonist of toll-like receptors TLR7 and TLR8, to enhance cancer immunotherapy [98]. In line with this, recently engineered microparticles (MPs) derived from alpha-fetoprotein (AFP)-overexpressing macrophages loaded with resiquimod (R848@M2pep-MPsAFP) were developed to target and reprogram M2-like TAMs into M1-like phenotypes to ameliorate tumour immunosuppressive microenvironments [99]. Additionally, R848@M2pep-MPsAFP induces stronger stem-like CD8^+^ T cell proliferation and differentiation to achieve a long-term immune surveillance to boost anti-PD-1 therapy in HCC [99]. In this respect, a clinical trial employing the RO7119929 agent, which is a TLR7 agonist, in combination with tocilizumab (anti-IL-6 receptor) has been performed (NCT04338685). However, combination therapy with a checkpoint inhibitor may be needed to leverage the proinflammatory potential of the RO7119929 agent to enhance the anti-tumour activity [100].

On the other hand, other strategies to enhance the anti-tumour responses of TAMs include restoring their phagocytic capacity by blocking the “don’t eat me” signal of CD47-SIRPα. CD47 expression in HCC cells is correlated with the poor overall survival of patients with HCC [101] and antibody-mediated targeting CD47 was shown to inhibit tumour growth in mouse models of HCC [102]. In addition, combination therapies of CD47 blockade with the anti-CD47 antibody and doxorrubicin have demonstrated an enhanced beneficial effect as compared to doxorrubicin in monotherapy [103]. Moreover, CD47 expression levels in HCC cells might serve as a prognostic marker of patients who might benefit from TACE treatment [101]. In this respect, anti-human SIRPα antibodies have been developed for the treatment of HCC and assessed in a clinical trial (NCT02868255), being the objective of this trial to harvest samples from patients with HCC (inflammatory ascites, HCC resections and blood samples) to evaluate SIRP-CD47 expression and the effect of the anti-hSIRP Ab on various cellular types.

Recent genetic engineering technologies, such as chimeric antigen receptor macrophages (CAR-M), have started to pave the way for using macrophages in adoptive cell therapies to treat solid tumours [104]. In vitro, CAR-Ms have demonstrated to have antigen-specific phagocytosis capacity and to promote tumour clearance. Of note, an infusion of CAR-Ms have been shown to prevent tumour progression in various solid tumour mouse models [104]. A phase I human study of adenovirally transduced autologous macrophages engineered to contain an anti-HER2 CAR in patients with HER2 overexpressing solid tumours in monotherapy and in combination with pembrolizumab is currently ongoing (NCT04660929). 

Regarding their potential applicability in HCC, a liver macrophage-targeting mRNA-laden lipid nanoparticle (LNP) was recently generated to produce CAR-Ms coexpressing glypican-3 (GPC3)-specific CAR, a glycoprotein attached and extensively upregulated in HCC tissues, and Siglec-G lacking immunoreceptor tyrosine-based inhibition motifs (Siglec- GΔITIMs). Siglec-G is expressed on the surface of macrophages and interacts with the anti-phagocytic signal protein CD24 upregulated in the tumour tissues of HCC patients. This interaction activates the ITIM motif in macrophages, which is known to restrict the phagocytic function of macrophages. Thus, this novel approach may help macrophages to identify and competitively bind to CD24 of HCC cells, augmenting the cellular phagocytosis by evading the activation of ITIMs, subsequently favouring the antigen cross-presentation of CAR-Ms [105]. In fact, mice treated with LNPs generating CAR-Ms as well as CD24-Siglec-G blockade are able to augment the phagocytic function of liver macrophages, reduce tumour burden and increase the survival of mice subjected to an orthotopic HCC model. 

**Table 4:** Preclinical studies and clinical trials targeting TAMs in HCC.

Molecular Target	Agent	Combination Therapy	Results	Clinical Trial Number	References
CCL2/CCR2 axis	CCR2 antagonist (RDC018)	N/A	Inhibits tumour growth and metastasis and prolongs the survival of mice.		[83]
	CCR2 antagonist (747)	Sorafenib (low-dose)	Enhances the therapeutic efficacy of low-dose sorafenib, elevating the numbers of intra-tumoural CD8+ T cells and increasing death of tumour cells.		[84]
CCL2/CCR2 and CCL5/CCR5 axis	CCR2/CCR5 inhibitor (BMS-813160)	Anti-PD1 mAb (Nivolumab)	Clinical trial Phase II (ongoing)	NCT04123379	
CSF1/CSFR1 axis	Gö6976, a protein kinase inhibitor, lenvatinib, or using a CSF-1R inhibitor (BLZ945)	Anti-PD1 therapy	Gö6976 or BLZ945 combined with anti-PD1 inhibit tumour growth. Lenvatinib and anti-PD1 exert synergistic anti-tumour effects and prolongs the survival of mice		[40,88]
	CSF1R inhibitor (PLX3397)	N/A	CSF1R blockade delays tumour growth by shifting the polarization of TAMs toward an M1-like phenotype.		[41]
	CSF1R inhibitor (PLX3397)	Anti-PD-L1	Blocking CSF1/CSF1R prevents TAM trafficking and enhances the efficacy of anti-PD-L1.		[42]
	Anti-CSF1R mAb (Cabiralizumab)	Anti-PD1 mAb (Nivolumab)	Clinical trial Phase II (ongoing)	NCT04050462	
PI3Kγ	TG100-115	Sorafenib	Higher anti-tumour efficiency than the free drug solutions.		[93]
	Pan-PI3K inhibitor (SF1126)	Anti-PD1 mAb (Nivolumab)	Clinical trial Phase I	NCT03059147	
C/EBPα	saRNA; MTL-CEBPA	Sorafenib	A marked reduction in tumour growth following MTL-CEBPA treatment is observed in preclinical mouse HCC models.		[95]
	saRNA; MTL-CEBPA	Sorafenib	Clinical trial Phase Ib. MTL-CEBPA causes radiologic regression of tumours in 26.7% of patients with HCC with an underlying viral etiology.	NCT02716012	[95]
	saRNA; MTL-CEBPA	Anti-PD1 mAb (Pembrolizumab)	Clinical trial Phase Ia/Ib (ongoing)	NCT04105335	
GSK3β	GSK3β inhibitor	Anti-PD1 mAb	Macrophage GSK3β-deficiency inhibits the progression of HCC and enhances the sensitivity of anti-PD1 immunotherapy.		[96]
RIPK3	RIPK3 inhibitor (GSK872)	N/A	Enhances M2 markers (CD206 and Arg1) and PPARs (Ppara and Pparg) in macrophages.		[97]
TLR7 and TLR8 agonists	R848@M2pep-MPsAFP	Anti-PD-1 mAb	R848@M2pep-MPsAFP efficiently reprograms M2-like macrophages and activates CD8+ T cells decreasing the tumour growth and prolonging the survival of mice improving the anti-tumour immune response of anti-PD-1 antibody.		[99]
	TLR7 agonist (RO7119929)	N/A	Clinical trial Phase I. Combination therapy with ICIs may be needed to enhance its anti-tumour activity.	NCT04338685	[100]
CD47-SIRPα	Anti-CD47 mAb	N/A	CD47 blockade inhibits tumour growth in mouse heterotopic and orthotopic models of HCC.		[102]
	Anti-CD47 mAb	Doxorubicin	Anti-CD47 Ab in combination with doxorubicin exerts maximal effects on tumour suppression in a patient-derived HCC xenograft mouse model, as compared to monotherapies alone.		[103]
	Anti-hSIRPα Ab	N/A	Clinical trial. Collection of human samples	NCT02868255	
CAR macrophages	LNP-mediated dual mRNA co-delivery of Siglec-GΔITIMs-expressing GPC3-specific CAR macrophages	N/A	LNP-engineered Siglec-GΔITIMs-expressing GPC3-specific CAR-Ms present augmented HCC-specific engulfment of macrophages, subsequently stimulating an adaptive anti-tumour immune response and preventing tumour growth in an orthotopic HCC mouse model.		[105]
	Anti-HER2 CAR-M (CT-0508) in patients with HER2 overexpressing solid tumours	Anti-PD1 mAb (Pembrolizumab)	Clinical trial Phase I (ongoing)	NCT04660929	

Abbreviations: Arg1, arginase 1; CAR, chimeric antigen receptor; CCL, C-C motif chemokine ligand; CCR, C-C motif chemokine receptor; CD, cluster of differentiation; C/EBPα, CCAAT/enhancer-binding protein alpha; CSF1, colony-stimulating factor 1; CSF1R, colony-stimulating factor 1 receptor; GPC3, glypican 3; GSK3β, glycogen synthase kinase 3β; HCC, hepatocellular carcinoma; ICI, immune checkpoint inhibitor; LNP, lipid nanoparticle; mAb, monoclonal antibody; PD-1, programmed cell death protein 1; PD-L1, programmed cell death ligand 1; PI3Kγ, phospatidylinositide 3-kinase γ; RIPK3, receptor interacting serine/threonine kinase 3; SIRPα, signal-regulatory protein α; TAMs, tumour-associated macrophages; TLR, toll-like receptor.

## 5. Future Perspectives and Conclusions

In cancer, the existence of a distinct microenvironment of each tumour contributes to the heterogeneity of macrophages [106]. Although many studies in HCC have defined distinct macrophage populations, this has been achieved by applying their own unique nomenclature, despite there being considerable overlap across the different studies [20,21,24,25,26,27,28,29,30]. In line with this, TAMs might derive primarily from circulating blood monocytes, even though evidence also suggests that KCs might also account for a small part of the total TAM pool of HCC [19]. Lineage tracing of TAMs in HCC can lead to a more thorough understanding of the complex nature of the TME. In future experiments, given the heterogeneity of TAMs, it will be important to describe their origin, surface markers, role and spatial distributions to identify the specific types being studied in each experiment. Moreover, as specific macrophage subsets assume distinct roles in HCC and anti-tumour immunity, avoiding simply classifying macrophages into M1 and M2 groups is important, and studies in the future should focus on carefully characterizing the great diversity of macrophage subpopulations.

TAMs can dynamically interact and crosstalk with HCC tumour cells modulating the development, growth and invasiveness of HCC cells by a great variety of mechanisms. In this regard, HCC tumour cells are known to secrete signalling molecules such as cytokines, chemokines and growth factors that are able to promote the transition of macrophages into TAMs acquiring a predominant M2-like phenotype. Most of the studies characterizing the secretome of HCC cells and the influence of the secreted factors on macrophages are based on in vitro models that do not recapitulate the great complexity and diversity of macrophages that are present in the TME of HCC tumours.

Recently, TAMs have emerged as a relevant cell type for targeted therapy in liver cancer and several preclinical and clinical therapeutic approaches have been developed aiming to deplete or reduce this population in HCC tumours. Nevertheless, pan-macrophage therapeutic strategies targeting all macrophages are often associated with a compensatory emergence of other immunosuppressive cell types and the presence of systemic toxicity, with limited therapeutic benefits. In this respect, reprogramming M2-like TAMs to acquire an immunostimulatory phenotype may represent a more effective strategy, in particular when this approach is combined with other treatments such as immune checkpoint inhibitors. Despite the fact that preclinical studies have demonstrated encouraging results, the use of animal models have some limitations, as they do not recapitulate the complex heterogeneity and full spectrum of TAM subtypes that are present in humans. At present, there are only a small number of clinical trials targeting TAMs. Nonetheless, their efficacy needs to be further assessed. 

Adoptive cell therapies using genetic engineered technologies such as CAR-M have also started to enter the field of cancer, also holding promise for the treatment of HCC. Accordingly, macrophages equipped with tumour antigen-specific CARs present increased anti-tumour activity. However, major challenges remain to be solved before their potential clinical application, including the selection of liver tumour cell-specific targets and their biosafety profiles. On the one hand, macrophages present a relatively weak proliferative capacity, which might affect the prolonged therapeutic benefit. Moreover, the high heterogeneity of HCC tumours might compromise the efficiency of CAR-Ms, as some HCC cells that do not express the target antigens may exist. In addition, although promising results might be obtained from experimental in vivo studies, further clinical trials in patients with a much more complex HCC heterogeneity are required to evaluate the efficiency of CAR-Ms. 

In the future, tailoring TAM-targeted therapies in combination with other therapeutic strategies may constitute a promising alternative treatment for patients with HCC. 

## Abbreviations

CAF: cancer-associated fibroblast; CAR-M, chimeric antigen receptor macrophage; CSF1, colony-stimulating factor 1; ECM, extracellular matrix; EMT, epithelial to mesenchymal transition; FDA, food and drug administration; HBV, Hepatitis B virus; HCC, Hepatocellular carcinoma; HCV, Hepatitis C virus; ICI; immune checkpoint inhibitor; IL, interleukin; KC, Kupffer cell; NAFLD, nonalcoholic fatty liver disease; mAb, monoclonal antibody; MDSC, myeloid-derived suppressor cells; MTA, molecular targeted agent; PD-L1, programmed cell death ligand 1; sc-RNA-seq, single-cell RNA-sequencing; SPP1, osteopontin; TAM, tumour-associated macrophage; TKI, tyrosine kinase inhibitor; TME, tumour microenvironment; VEGF, vascular endothelial growth factor.
